# Preventive Antibiotic Therapy in the Placement of Immediate Implants: A Systematic Review

**DOI:** 10.3390/antibiotics11010005

**Published:** 2021-12-22

**Authors:** Angel-Orión Salgado-Peralvo, Juan-Francisco Peña-Cardelles, Naresh Kewalramani, María-Victoria Mateos-Moreno, Álvaro Jiménez-Guerra, Eugenio Velasco-Ortega, Andrea Uribarri, Jesús Moreno-Muñoz, Iván Ortiz-García, Enrique Núñez-Márquez, Loreto Monsalve-Guil

**Affiliations:** 1 Department of Stomatology, University of Seville, 41009 Seville, Spain; alopajanosas@hotmail.com (Á.J.-G.); evelasco@us.es (E.V.-O.); je5us@hotmail.com (J.M.-M.); ivanortizgarcia1000@hotmail.com (I.O.-G.); enrique_aracena@hotmail.com (E.N.-M.); lomonsalve@hotmail.es (L.M.-G.); 2 Science Committee for Antibiotic Research of Spanish Society of Implants (SEI—Sociedad Española de Implantes), 28020 Madrid, Spain; juanfranciscopenacardelles@gmail.com (J.-F.P.-C.); k93.naresh@gmail.com (N.K.); 3 Department of Basic Health Sciences, Rey Juan Carlos University, 28922 Madrid, Spain; auribarride@gmail.com; 4 Department of Nursery and Stomatology, Rey Juan Carlos University, 28922 Madrid, Spain; 5 Department of Clinical Specialties, Faculty of Dentistry, Complutense University of Madrid, 28040 Madrid, Spain; mateosmoreno80@hotmail.com

**Keywords:** immediate implants, antibiotic prophylaxis, preventive antibiotics, antibiotic, early failure, dental implant complications

## Abstract

Immediate implants present a high risk of early failure. To avoid this, preventive antibiotics (PAs) are prescribed; however, their inappropriate administration leads to antimicrobial resistance. The present study aims to clarify whether the prescription of PAs reduces the rate of early failure of immediate implants and to establish guidelines to avoid the overprescription of these drugs. An electronic search of the MEDLINE database (via PubMed), Web of Science, Scopus, LILACS and OpenGrey was carried out. The criteria described in the PRISMA^®^ statement were used. The search was temporarily restricted from 2010 to 2021. The risk of bias was analysed using the SIGN Methodological Assessment Checklist for Systematic Reviews and Meta-Analyses and the JBI Prevalence Critical Appraisal Tool. After searching, eight studies were included that met the established criteria. With the limitations of this study, it can be stated that antibiotic prescription in immediate implants reduces the early failure rate. Preoperative administration of 2–3 g amoxicillin one hour before surgery followed by 500 mg/8 h for five to seven days is recommended. It is considered prudent to avoid the use of clindamycin in favour of azithromycin, clarithromycin or metronidazole in penicillin allergy patients until further studies are conducted.

## 1. Introduction

Traditionally, implant placement was performed several months to a year after tooth extraction [1]. However, after tooth loss, the lack of stimuli in the residual bone causes a decrease in bone and trabecular density, with loss of bone volume [2] that may hinder the subsequent placement of dental implants [3]. More specifically, there is resorption of the alveolar process of 5–7 mm in the horizontal or buccolingual direction (about 50% of the initial width) [4] and a reduction of 2–4.5 mm in the vertical or apicocoronal direction [5,6] between 6–12 months following tooth exodontia [4,5,6]. Inevitably, these changes in the bone lead to secondary soft tissue alterations that may affect the aesthetics of the future implant-supported restoration [7]. The placement of immediate implants, i.e., at the time of tooth extraction, reduces these tissue changes and, in the case of a provisional restoration with immediate prosthetic loading, eliminates the use of interim removable prostheses with the disadvantages that this may involve, offering optimal aesthetics during the healing period [8]. They also reduce morbidity and the number of appointments required, resulting in a shorter overall treatment time [8,9], improving patient experience and satisfaction [10].

Except for dental trauma, in all other clinical circumstances, exodontia is performed for infectious reasons such as periodontal disease, caries and/or fissures/fractures resulting in bacterial contamination. During the post-extraction healing process, these infectious processes usually resolve, however, the presence of the previous infection increases the inflammatory response, resulting in increased bone resorption that can lead to loss of implant stability and early immediate implant failure [11], i.e., during the osseointegration process [12,13]. In this regard, immediate implant placement is considered a risk factor for early failure, with failure rates of 5.1% versus (vs.) 1.1% for delayed placements (from six months after extraction) and, in turn, immediate implants placed in sites with periapical infections fail up to three times more [14,15].

To avoid these complications, the prescription of preventive antibiotics (PAs) was incorporated into implant placement protocols. However, due to the indiscriminate and sometimes inappropriate use of these drugs, we are entering a post-antibiotic era in which many common infections and minor injuries will again become life-threatening due to a loss of efficacy of these drugs and, even if new ones are developed if current prescribing patterns and population habits are not changed, resistance will continue to pose a serious threat to public health [16]. Therefore, preventive antibiotic therapy in dental implant procedures is currently a controversial issue [17]. At the 4th European Association for Osseointegration (EAO) Consensus Conference in 2015 [18], it was determined that in complex cases—such as immediate implant placement—a beneficial effect of PAs cannot be excluded. However, there are currently no established protocols on how to prescribe them in such procedures.

The present study aims to evaluate whether the prescription of PAs in the placement of immediate implants reduces the rate of early implant failure and, if so, which regimen is recommended based on the available scientific evidence.

## 2. Material and Methods

This systematic review was structured according to the Preferred Reporting Items for Systematic Reviews and Meta-Analyses (PRISMA^®^) statement [18].

### 2.1. Focused Question

The study aimed to answer the following PICO (P = patient/problem/population; I = intervention; C = comparison; O = outcome) question based on the PRISMA^®^ guidelines (Table 1):

In patients undergoing immediate implant placement, with or without infection of the tooth to be extracted, does the prescription of PAs decrease early implant failure rates compared to not prescribing them?

The secondary objective was to determine the type of PAs, dose and posology recommended in these cases according to the available scientific evidence.

### 2.2. Clinical Relevance

Immediate implants are becoming more and more widely used due to the advantages described above. Currently, there are only clear protocols regarding the prescription of PAs in the placement of implants without anatomical constraints [19] and in bone augmentation with one- or two-stage implant placement [20], but not in other procedures, such as the placement of immediate implants. Therefore, it is important to establish protocols to avoid indiscriminate prescription of these drugs to reduce and/or avoid antimicrobial resistance.

### 2.3. Eligibility Criteria

Before starting, inclusion and exclusion criteria were defined for the resulting articles:

#### 2.3.1. Inclusion Criteria

Included studies were: (a) studies conducted in humans; (b) articles published in English; (c) meta-analyses; (d) systematic reviews; (e) clinical trials; (f) controlled clinical trials; (g) randomized clinical trials (RCTs); (h) multicenter studies; (i) observational studies; (j) comparative studies; and (k) articles analysing the influence of PAs on early failure rates of immediate implant placement in sites with or without previous infection.

#### 2.3.2. Exclusion Criteria

The exclusion criteria determined the exclusion of the following: (a) experimental laboratory studies; (b) animal studies; (c) studies whose main topic was not the prescription of PAs in immediate dental implants placement; (d) duplicate articles; (e) books or chapters of books; (f) letters to the Editor; (g) commentaries; (h) case reports; and (i) narrative literature reviews.

### 2.4. Information Sources and Search Strategy

A comprehensive search of the literature was conducted in the following databases: PubMed/Medline, Web of Science, Scopus and LILACS. A search for unpublished studies (grey literature) was conducted on the OpenGrey database. In addition, we examined the bibliographic references of the selected articles for publications that did not appear in the initial search and might be of interest.

The search was performed by two independent researchers (A.-O.S.-P. and J.-F.P.-C.). The search was temporarily restricted from 2010 to 2021 and was updated to 14 November 2021.

MeSH (Medical Subject Headings) terms, keywords and other free terms were used with Boolean operators (OR, AND) to combine searches: ((((((immediate implant placement[Title/Abstract]) OR (immediate implant[Title/Abstract])) OR (immediate implants[Title/Abstract])) OR (immediate implantation[Title/Abstract]) OR (((((dental implant[MeSH Terms]) OR (dental implants[Title/Abstract])) OR (dental implantation[Title/Abstract])) OR (oral implantology[Title/Abstract])) OR (dental implantology[Title/Abstract]))) AND (((fresh extraction socket[Title/Abstract]) OR (tooth extraction[Title/Abstract])) OR (tooth socket[Title/Abstract])))) AND (((((((((antibiotic prophylaxis[MeSH Terms]) OR (antibiotics[MeSH Terms])) OR (preventive antibiotics[Title/Abstract])) OR (preventive antibiotic[Title/Abstract])) OR (amoxicillin[MeSH Terms])) OR (amoxicillin clavulanic acid[MeSH Terms])) OR (clindamycin[MeSH Terms])) OR (azythromycin[MeSH Terms])) OR (erythromycin[MeSH Terms])). The same keywords were used for all search platforms followed the syntax rules for each database.

### 2.5. Study Records

Two researchers (A.-O.S.-P. and J.-F.P.-C.) independently compared the results to ensure completeness and removed duplicates. Then, the full title and abstracts of the remaining papers were screened individually. Finally, full-text articles to be included in this systematic review were selected according to the criteria described above. Disagreements over eligible studies to be included were discussed with a third reviewer (N.K.) and a consensus was reached. The reference list of the included studies was also reviewed for possible inclusion.

### 2.6. Risk of Bias

Data collection was conducted using a predetermined table designed in advance of the assessment of the resulting articles. Two independent reviewers (A.-O.S.-P. and J.-F.P.-C.) evaluated the methodological quality of eligible studies following the SIGN Methodological Assessment Checklist for Systematic Reviews and Meta-Analyses [21] developed by Healthcare Improvement Scotland. This tool assesses (1) the internal validity of the studies by analysing 12 items, and (2) the overall assessment of the study, determining the methodological quality of the systematic reviews and/or meta-analyses included, classifying them as “high quality (++)” (majority of criteria met. Little or no risk of bias); “acceptable (+)” (most criteria met. Some flaws in the study with an associated risk of bias); “low quality (−)” (either most criteria not met, or significant flaws relating to key aspects of the study design); or “reject (0)” (poor quality study with significant flaws. Wrong study type. Not relevant guideline). On the other hand, the Joanna Briggs Institute (JBI) Prevalence Critical Appraisal Tool [22], which incorporates 10 domains, was used to analyse the remaining studies. The studies were classified as low-quality assessment studies (0–5 domains), or as high-quality assessment studies (6–10 domains).

## 3. Results

### 3.1. Study Selection

The search strategy resulted in 366 results, of which 361 remained after removing the duplicates. Then, two independent researchers (A.-O.S.-P. and J.-F.P.-C.) reviewed all the titles and abstracts and excluded 349 that were outside the scope of this review. Thus, we obtained 17 potential references. After reading the full text of those 17 papers, 12 were discarded for referring to the prescription of PAs in ordinary surgical site conditions, i.e., on native bone (*n* = 2), for not comparing the prescription of PAs vs. not prescribing them or vs. prescribing another antibiotic regimen (*n* = 8), for being a narrative literature review (*n* = 1) or for having an insufficient sample size (*n* = 1).

**Figure 1:**
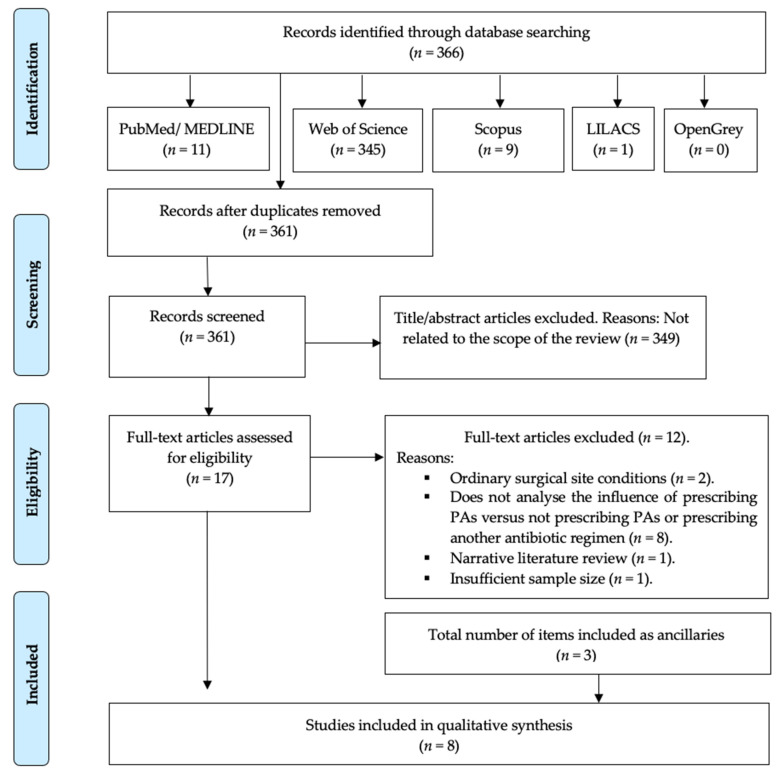
PRISMA^®^ flow diagram of the search processes and results.

When analysing the references of the reviewed articles, three articles were included as ancillaries. Therefore, 8 studies were included in our systematic review (Figure 1).

### 3.2. Study Characteristics

Of the eight studies included, five were systematic reviews [11,22,23,24,25], one of them was also a meta-analysis [26], one was an RCT [27] and another one, a retrospective, non-interventional open cohort study [28]. The type of antibiotic and the regimens used in the included studies were not provided by all authors. Of those that did provide these data, it can be concluded that they were very heterogeneous [11,24,26,29,30], reflecting a lack of consensus.

The main findings of each investigation are described below (Table 2):

Cosyn et al. [26] (2019) reported immediate implant failure rates of 5.1% vs. 1.1% for delayed placements, i.e., 6 months after tooth extraction, with these differences being significant (Relative Risk [RR] = 0.96; *p* = 0.02). All failures were early. When analysing the prescribed PAs regimens, a trend towards lower survival of immediate implants was observed when PAs were not administered postoperatively (RR = 0.93). On the other hand, in both implant placement protocols (immediate vs. delayed) healing was adequate, except in one study [29] where they found a fivefold higher risk of surgical wound complications in immediate implants (26.1% vs. 5.3%, respectively), which could be because immediate implants often require simultaneous guided bone regeneration (GBR).

Lee et al. [25] (2015) concluded that there is no specific protocol regarding the antibiotic regimen to be used in immediate implant placement, but they recognised the need to prescribe PAs in these cases.

Chrcanovic et al. [11] (2015) conducted a systematic review including studies that investigated the prognosis of immediate implants in infected sites. It included animal (*n* = 7) and human (*n* = 21) studies, none of which compared immediate implant placement with and without the prescription of PAs, so there is no test group with which to compare the results. If only human studies are considered and all cases of immediate implants are included, without distinction between the previous pathology or not, the failure rate is 1.7%. The total duration of PA therapy in the different studies included was 6–14 days. The most frequent regimen was perioperative, although some studies carried out only pre- or postoperative PAs.

Álvarez-Camino et al. [24] (2013) highlighted the need to prescribe PAs in immediate implants in infected sites, however, they did not recommend a specific guideline.

Lang et al. [22] (2012) conducted a systematic review including 46 studies, of which 33 prescribed PAs: four carried out preoperative PAs (*n* = 244 implants) and, in 15 studies, only postoperative antibiotic administration was recommended, with a duration of five to seven days (*n* = 935 implants). The remaining 14 studies prescribed perioperative PAs (one preoperative dose followed by five to seven days postoperatively) (*n* = 665 implants). To determine the failure rates of immediate implants placed under each regimen, they performed a multivariate analysis using a fixed-effect Poisson regression model, considering the preoperative prescription as the reference. In this way, they calculated the annual failure rate of implants placed under preoperative prophylaxis at 1.87%; postoperative at 0.51% and, under perioperative guidelines, at 0.75%, these differences being significant (*p* = 0.002). Therefore, a preoperative prophylactic single-dose regimen is not sufficient to maintain bacterial levels below the critical threshold during the healing period, but prescribing PAs 5–7 days post-surgery may help to prevent complications that could lead to implant failure.

Waasdorp et al. [23] (2010) did not elaborate on the type of antibiotic regimen recommended but, despite stating that there is controversy about its use, they recommend prescribing PAs in immediate implants in infected sites. Antibiotic regimens were very heterogeneous, with treatment durations ranging up to 31 days. Failure rates ranged from 0–8%. In studies where antibiotics were prescribed postoperatively, the failure rate was 0–2.3% (*n* = 4), in those prescribed preoperatively 8% (*n* = 1) and perioperatively 0–2.6% (*n* = 2).

Esposito et al. [29] (2010) conducted a multicentre RCT involving 506 patients, in which they studied the effect on implant success and postoperative complications of prescribing amoxicillin 2 g, 1 h preoperatively vs placebo. 19.6% of patients (*n* = 99) received immediate implants (test group = 60 implants; control group = 76 implants), with an implant failure rate of 9% vs. 2% in those patients who received delayed implants (*p* < 0.001). Specifically, five immediate implants failed in the test group (8.3%) and 12 in the control group (15.8%), with no significant difference between the two groups (*p* = 0.48).

French et al. [30] (2016) compared different types of PAs to determine whether self-declared penicillin-allergic (SRPA) patients have a higher rate of implant failure. Specifically, these authors prescribed amoxicillin 2 g, 1 h before surgery, followed by 250 mg/8 h, for seven days and, in SRPA-patients, 600 mg, 1 h preoperatively, followed by 150 mg, 6 h, for seven days. Of the 5,576 implants placed, 687 were immediate (12.3%), observing a failure rate of 1.7% (*n* = 12). Of these, 1% failed in non-allergic patients (6/630) and 10.5% in SRPA-patients (6/57) (*p* < 0.001)

### 3.3. Risk of Bias within Studies

Risk of bias and study quality analyses were performed independently by two review authors (A.-O.S.-P. and J.-F.P.-C.). Using the predetermined SIGN Methodological Assessment Checklist for Systematic Reviews and Meta-Analyses [21] developed by Healthcare Improvement Scotland, three studies were found to be of high quality [11,25,28], two of acceptable quality [23,25] and one of low quality [24]. Table 3 shows a more detailed description of the articles included. On the other hand, using the predetermined 10 domains for the methodological quality assessment according to JBI Prevalence Critical Appraisal Tool [30] for the two remaining studies, we determined that both papers have a high-quality assessment. Even though French et al. [28] (2016) is a retrospective study, it has better quality than the Esposito et al. [27] (2010) RCT (Table 4).

## 4. Discussion

Immediate implants placed in sites with apical pathology fail up to three times more often than those placed in the absence of pathology [14] because of the potential for implant contamination during the initial healing period due to the presence of pathogenic bacteria [11]. Bacteroides species can colonise periapical lesions while remaining encapsulated in polysaccharides that enhance their virulence and survival in mixed infections [31]. In particular, *Tannerella forsythia* persists asymptomatically in periradicular endodontic lesions and survives at bone level encapsulated after tooth extraction and can infect immediate implants [32]. In this regard, Ayangco & Sheridan [33] described three cases of patients with a history of failed endodontic treatment and subsequent apicectomy resulting in extractions of the affected teeth (*n* = 4). They subsequently placed implants 9–16 weeks post-extraction, despite which retrograde peri-implantitis occurred due to the permanence of bacteria (cyst/granuloma) at bone level. These conclusions are supported by other authors who postulate that, despite extraction of a tooth with apical pathology and thorough curettage of the tooth socket, bacteria persist and can reactivate and cause infection in subsequent implant treatment [34]. In this regard, Kassolis et al. [35] described the presence of biofilm-forming regions and necrotic alveolar bone in edentulous jaws up to one year after tooth extraction as a risk factor for early dental implant failure. Despite this, acute or chronic endodontic infections are usually of a mixed type, with anaerobic species such as *Fusobacterium*, *Prevotella*, *Prophyromonas*, *Actinomyces*, *Streptococcus* and *Peptostreptococcus* predominating, commonly located in the root canal area [36], so that after extraction of the contaminated tooth, the microorganisms usually disappear [37].

Animal model studies [38] and studies conducted in humans [23,25] suggest that after adequate debridement and prescription of systemic PAs, an adequate bone remodelling process can be achieved around immediate implants placed in infected sites. Also, chemical methods in combination with mechanical methods—such as debridement—have been proposed for the decontamination of postextraction alveoli. In this regard, Montoya-Salazar et al. [39] compared clinical and radiological results after placing 36 immediate implants (18 were placed in non-infected sites [control group] and 18 in infected sites after being debrided, curetted, cleaned with 90% hydrogen peroxide, and irradiated with yttrium-scandium-gallium-garnet (Er, Cr:YSGG) laser [test group]). All patients were administered amoxicillin 500 mg/8 h, from four days before to six days after surgery. Patients with penicillin sensitivity received 300 mg clindamycin every 8 h for the same period (10 days). All patients experienced a 100% survival rate at 24 months follow-up (*p* = 0.720). Immediate implant failure rates of 0–9% were reported in the included studies. In general, higher failure rates in this type of implant placement protocols have been reported in the literature, although authors such as Esposito et al. [40] (2017) studied complications after immediate, immediate-delayed (six weeks) and delayed (four months) insertion of dental extractions on single implants, describing failure rates of 6% for immediate implants, 6.2% for immediate-delayed implants and 1.6% for delayed implants (*p* = 0.369).

Despite this, in the opinion of the authors, immediate implants should, in any case, be approached as if the tooth to be extracted is chronically infected, as these are sometimes asymptomatic without apparent clinical signs of infection, which can lead to implant loss [39,41]. With the limitations of this study, it can be stated that the prescription of PAs in these cases reduces the rate of early failure of implants. Some authors have linked the use of clindamycin in immediate implants in penicillin-allergic patients as a risk factor for early failure [40,42]. Specifically, they found a 5.7 [43] to 10-fold [28] increased risk compared to non-allergic patients prescribed with amoxicillin. These authors explained these findings with an increased risk of infection in allergic patients [28,43], however, no specific diagnostic test was performed before inclusion in the studies, i.e., patients self-reported to be allergic. In clinical practice, 10–20% of patients report an allergy or reaction to penicillins, however, these are rarely hypersensitivity or immunoglobulin E-mediated reactions [44,45,46]. Furthermore, studies have shown that 80–99% of these patients may no longer be considered allergic after testing [47,48,49]. Therefore, penicillin allergy per se cannot be considered as a risk factor for early failure so far. Instead, it seems more plausible that alternative antibiotics, such as in this case clindamycin, present a suboptimal efficacy [50,51,52,53,54] or a negative influence on osseointegration [55].

Scientific evidence has shown that administration of a single preoperative dose of antibiotics is not sufficient to maintain bacterial levels below the critical threshold during the healing period, but prescribing them five to seven days postoperatively may help prevent postoperative infection that can lead to failure of osseointegration [22]. Jofre et al. [56] (2012) established a protocol for immediate implant placement in infected sites in which, despite not recommending a specific type of PA and dosage, they recommended perioperative prescription.

There is no evidence to recommend a specific type and dose of antibiotic in these cases, so until further studies are conducted it is necessary to recommend a specific guideline based on the conclusions drawn from the available studies to ensure that professionals follow clear recommendations and thus reduce the risk of early implant failure and the probability of the emergence of antimicrobial resistance. Therefore, it is considered prudent to apply the recommendations established by the European Society of Endodontology [57] (2018) given the nature of the microbiota to be avoided, which advise the administration of antibiotics with a loading dose followed by a maintenance dose. The present investigation suggests applying the loading dose advised by a recent network meta-analysis for conventional implant placement [19], which coincides with that suggested by a recent systematic review on the prescription of PAs in GBR with 1- or 2-stage implant placement [20], which recommends 2–3 g of amoxicillin one hour before surgery, followed by the maintenance dose for five to seven days, i.e., 500 mg/8 h for five to seven postoperative days. In the case of confirmed true penicillin allergy, first-line alternatives would be clindamycin 600 mg one hour preoperatively, followed by 300 mg/6 h for five to seven days, however, given the associated higher early failure rates, it would be prudent to avoid its use until further studies are conducted. Other alternatives are azithromycin 500 mg one hour before [42] followed by 250 mg/24 h, for five to seven days; clarithromycin 500 mg one hour before followed by 250 mg/12 h, for five to seven days; and metronidazole 1 g one hour preoperatively followed by 500 mg/6 h, for five to seven days (Table 5).

### Strengths and Limitations

This systematic review presents several strengths, such as the searching process of studies, data extraction and risk analysis bias performed in duplicate, which determined a high overall quality of the included studies.

Nonetheless, the present systematic review has limitations, such as the shortage of studies with control groups that allow a comparison between the groups, which is why the external validity of the results of this review should be confirmed with future studies.

## 5. Conclusions

With the limitations of this study, it can be stated that antibiotic prescription in immediate implants reduces the early failure rate. Preoperative administration of 2–3 g of amoxicillin one hour before surgery followed by 500 mg/8 h for five to seven days is recommended. In the case of penicillin allergy, until further studies are conducted, it is considered prudent to avoid prescribing clindamycin. In these cases, it is recommended to prescribe azithromycin 500 mg one hour before, followed by 250 mg/24 h, for five to seven days, clarithromycin 500 mg one hour before followed by 250 mg/12 h, for five to seven days, or metronidazole 1 g one hour before surgery, followed by 500 mg/6 h, five to seven days postoperatively. Immediate implants placed in sites with or without infection of the tooth to be extracted should be treated as if they have an infectious pathology due to the possibility of subclinical infection.

## Figures and Tables

**Table 1:** Breakdown of the “PICO” question.

Component	Description
P (problem/population)	Patients undergoing immediate DI ^1^ placement, with or without the presence of chronic infection of the tooth to be extracted
I (intervention)	PAs ^2^ on the day of surgery and/or extended postoperatively
C (comparison)	Not prescribing PAs Prescribing a placebo Other antibiotics or antibiotic regimens Same antibiotic with different dosage/duration
O (outcome)	DI failure Postoperative infection
PICO question	In patients undergoing immediate implant placement, with or without infection of the tooth to be extracted, does the prescription of PAs decrease early implant failure rates compared to not prescribing them?

^1^ DI, dental implant; ^2^ PAs, preventive antibiotics.

**Table 2:** Results of included studies. Data on immediately placed implants were included.

Author(s)/Year	Type of Study	No. ^1^ Patients/No. Immediate DI ^2^	Immediate DI Failure Rate (%)	Follow-Up (Months)	Conclusions
Cosyn et al. [26] (2019)	SR ^3^ and M-A ^4^	232/ 233	5.1	12–96	Tendency for lower survival of immediate implants in the absence of PostOp ^5^ PAs ^6^
French et al. [28] (2016)	Cohort study	UNS ^7^/ 687	1.7	120	The failure rate of immediate DI in SRPA ^8^ patients prescribed with clindamycin is 10 times higher than in the group prescribed with amoxicillin
Lee et al. [25] (2015)	SR	UNS/ 89	UNS	12–120	In favour of prescribing PAs in infected sites. It is not possible to recommend a guideline
Chrcanovic et al. [11] (2013)	SR	1259/ 1735	1.7	3–297	The most frequent pattern was PeriOp ^9^. It is not possible to draw a conclusion on the use of PAs in cases.
Álvarez-Camino et al. [24] (2013)	SR	NA ^10^	UNS	UNS	In favour of prescribing PA in infected sites. It is not possible to recommend a guideline.
Lang et al. [22] (2012)	SR	2073/ 2908	PreOp ^11^ PAs = 1.87 PostOp PAs = 0.51 PeriOp PAs = 0.75	56	PreOp prophylaxis is not sufficient, however, for 5–7 days PostOp may help prevent PostOp infections
Waasdorp et al. [23] (2010)	SR	186/ 324	0–8.0	7–72	They recommend prescribing PAs for immediate implants in infected sites
Esposito et al. [27] (2010)	RCT ^12^	99/ 136	9.0%	4	They found no evidence that 2 g amoxicillin 1 h PreOp reduces early failure in immediate implants vs. placebo

^1^ No, number; ^2^ DI, dental implants; ^3^ SR, systematic review; ^4^ M-A, meta-analyses; ^5^ PostOp, postoperative; ^6^ PAs, preventive antibiotics; ^7^ UNS, unspecified; ^8^ SRPA, self-reported penicillin allergy; ^9^ PeriOp, perioperative; ^10^ NA, not assessable; ^11^ PreOp, preoperative; ^12^ RCT, randomized clinical trial.

**Table 3:** SIGN Methodological Assessment Checklist for Systematic Reviews and Meta-Analyses [21].

Items	Cosyn et al. [26] (2019)	Lee et al. [25] (2015)	Chrcanovic et al. [11] (2013)	Alvarez-Camino et al. [24] (2013)	Lang et al. [22] (2012)	Waasdorp et al. [23] (2010)
Section 1: Internal Validity						
The research question is clearly defined, and the inclusion/exclusion criteria must be listed in the paper	^ 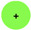 ^	^ 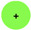 ^	^ 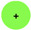 ^	^ 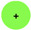 ^	^ 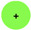 ^	^ 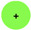 ^
A comprehensive literature search is carried out	^ 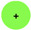 ^	^ 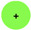 ^	^ 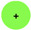 ^	^ 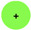 ^	^ 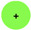 ^	^ 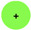 ^
At least two people should have selected studies	^ 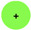 ^	^ 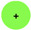 ^	^ 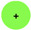 ^	^ 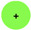 ^	^ 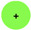 ^	^ 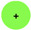 ^
The status of publication was not used as inclusion criterion	^ 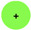 ^	^ 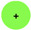 ^	^ 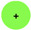 ^	^ 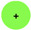 ^	^ 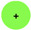 ^	^ 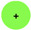 ^
The excluded studies are listed	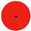	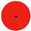	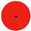	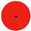	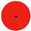	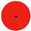
The relevant characteristics of the included studies are provided	^ 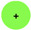 ^	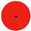	^ 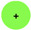 ^	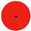	^ 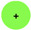 ^	^ 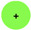 ^
The scientific quality of the included studies was assessed and reported	^ 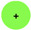 ^	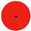	^ 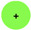 ^	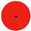	^ 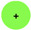 ^	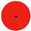
Was the scientific quality of the included studies used appropriately?	^ 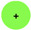 ^	^ 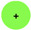 ^	^ 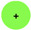 ^	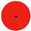	^ 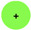 ^	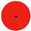
Appropriate methods are used to combine the individual study findings	^ 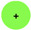 ^	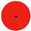	^ 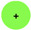 ^	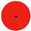	^ 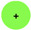 ^	^ 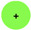 ^
The likelihood of publication bias was assessed appropriately	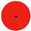	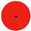	^ 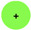 ^	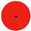	^ 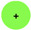 ^	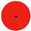
Conflicts of interest are declared	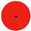	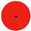	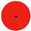	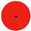	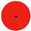	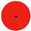
Section 2: Overall Assessment of the Study						
Are the results of this study directly applicable to the patient group targeted by this guideline?	^ 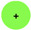 ^	^ 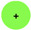 ^	^ 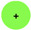 ^	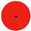	^ 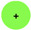 ^	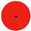
What is your overall assessment of the methodological quality of this review?	High Quality ^1^	Acceptable ^2^	High Quality	Low Quality ^3^	High Quality	Acceptable

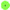
—Yes;
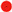
—No; ^1^ High quality, majority of criteria met. Little or no risk of bias; ^2^ Acceptable, most criteria met. Some flaws in the study with an associated risk of bias; ^3^ Low quality, either most criteria not met, or significant flaws relating to key aspects of study design.

**Table 4:** JBI Critical Appraisal Tool for studies reporting prevalence data [30].

Items	French et al. [28] (2016)	Esposito et al. [27] (2010)
1. Was the sample representative of the target population?	^ 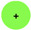 ^	^ 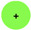 ^
2. Were study participants recruited in an appropriate way?	^ 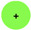 ^	^ 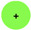 ^
3. Was the sample size adequate?	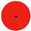	^ 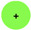 ^
4. Were the study subjects and setting described in detail?	^ 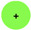 ^	^ 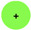 ^
5. Is the data analysis conducted with sufficient coverage of the identified sample?	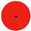	^ 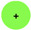 ^
6. Were objective, standard criteria used for measurement of the condition?	^ 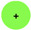 ^	^ 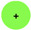 ^
7. Was the condition measured reliably?	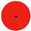	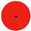
8. Was there appropriate statistical analysis?	^ 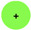 ^	^ 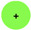 ^
9. Are all the important cofounding factors/subgroups/differences identified and accounted for?	^ 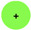 ^	^ 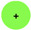 ^
10. Were subpopulation identified using objective criteria?	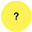	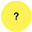

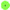
—Yes;
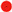
—No;
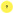
—Unclear.

**Table 5:** Recommended doses in the placement of immediate implants with or without the presence of chronic infection of the tooth to be extracted.

Antibiotic		PreOp ^1^ Dose (1 h ^3^ before)	PostOp ^2^ Dose (5–7 days)
Amoxicillin		2–3 g	500 mg/8 h
Beta-lactam antibiotic allergy	Clindamycin	600 mg ^4^	300 mg/6 h
	Azithromycin	500 mg	250 mg/24 h
	Clarithromycin	500 mg	250 mg/12 h
	Metronidazole	1 g ^5^	500 mg/6 h

^1^ PreOp, preoperative; ^2^ PostOp, postoperative; ^3^ h, hour(s); ^4^ mg, milligrams; ^5^ g, grams.

## Data Availability

Data available in a publicly accessible repository.
